# Optimization of Critical Parameters for Carbodiimide Mediated Production of Highly Modified Chitosan

**DOI:** 10.3390/polym13162702

**Published:** 2021-08-13

**Authors:** Henrik-Alexander Christ, Yannick Bourgat, Henning Menzel

**Affiliations:** Institute for Technical Chemistry, Braunschweig University of Technology, Hagenring 30, 38106 Braunschweig, Germany; h.christ@tu-braunschweig.de (H.-A.C.); y.bourgat@tu-braunschweig.de (Y.B.)

**Keywords:** arylazide, chitosan, functionalization, carbodiimide chemistry

## Abstract

An optimization of the 1-ethyl-3-(3-dimethylaminopropyl) carbodiimide and hydroxy benzotriazole mediated conjugation of the polysaccharide chitosan with functional carboxylic acids was shown. Optimal parameters that enable resource-efficient synthesis of highly functionalized chitosan were identified. In particular, use of only catalytic instead of stoichiometric amounts of hydroxy benzotriazole and tight control of pH in reaction mixture resulted in highly efficient incorporation of the desired moieties as side chains in chitosan. As a result, the model reactant 4-azidobenzoic acid was incorporated resulting in a degree of substitution of over 30% with very high coupling efficacy of up to 90%. Similar results were obtained with other carboxylic acids such as methacrylic acid, 3-(2-furyl) propionic acid and 3-maleimido propionic acid, highlighting the broad applicability of our findings for the functionalization of chitosan.

## 1. Introduction

Chitosan (CS) is a polysaccharide consisting of β-1-4-linked *N*-acetyl-glucosamine (GlcNAc) and glucosamine (GlcN) repetition units. It is derived from chitin, a natural polymer occurring in insects, crustacean, and fungi. Shells of shrimps and lobsters are currently the main biological source for chitin. Furthermore, fungi are another source of chitin that can be used for production of chitosan. Demineralization and deacetylation of chitin from marine organisms implicates high economic and ecological costs [1]. In contrast, chitin produced from fungal biomass represents a more reliable and ecofriendly alternative. Furthermore, the chitosan derived from the different sources typically have different properties such as degree of acetylation and molecular weight.

CS is derived by deacetylation of chitin and is of greater commercial interest because of its superior properties such as high solubility under aqueous acidic conditions, its biocompatibility, biodegradability, osteo-compatibility, antimicrobial and hemostatic activity as well as mucoadhesive properties [2]. Therefore, areas of application for CS range from biotechnology to pharmaceutical [2] and food industry [3,4] uses. Hence, grafting new functions to the chitosan chain is a pathway for further extending its possible applications. Examples for functional groups grafted to CS are: pH-indicators [5], click-chemistry ligands [6,7], DNA-nucelotides [2], amino acids [8,9], polymer chains (PLGA [10], PEO [8,9,11]), affinity ligands [12,13,14], antioxidant-enhancers [3,4,15] as well as moieties for crosslinking [8], photocrosslinking [16,17,18] and metal ion chelatization [15,19].

Almost all these modifications of CS have been accomplished by reacting primary amine groups present in the GlcN units of CS with carboxylic acid functions of the desired molecules. The amide forming reaction is typically carried out by use of coupling reagents to activate the carboxylic acid and to facilitate mild reaction conditions [20]. Most commonly used reagents for amide forming reactions of CS or other water-soluble biopolymers at aqueous conditions are either the triazine-based 4-(4,6-dimethoxy-1,3,5-triazin-2-yl)-4-methylmorpholinium chloride (DMT-MM) [16,21] or the carbodiimide-based 1-ethyl-3-(3-dimethylaminopropyl) carbodiimide (EDC) [2,3,4,5,6,9,11,13,18]. Both reagents form water-soluble side-products that can be easily separated from the desired biopolymers by dialysis [20]. EDC has, when compared with DMT-MM, specific advantages such as increased stability against organic solvents (e.g., dimethyl sulfoxide (DMSO)), which are necessary when conjugating a poorly water-soluble carboxylic acid, such as 4-azidobenzoic acid (Az) used here [6,22]. However, reactions with EDC are typically very sensitive to pH and therefore demand tight control in that regard [23,24]. In this respect it should also be noted that using DMSO as a cosolvent changes pKa of reagents and therefore influences pH in reaction mixture [25]. These facts present typical challenges from organic chemistry, but are sometimes neglected in the case of polymer analogous reactions [4,5,6,8,11,23].

Typically, additives are used for EDC mediated reactions to reduce epimerization, prevent side reactions, and therefore increase reaction efficacy. Despite the existence of both more efficient [20] and safer [26] alternatives, 1-hydroxybenzotriazol (HOBt) has so far often been the additive of choice for conjugation of various carboxylic acids to CS. However, the use of stoichiometric amounts of HOBt in most published conjugation experiments is associated with an insufficient degree of substitution (DS), low coupling efficacy (CE) and undesirable high reagent consumption [2,3,4,5,6,8,9,10,11,15,19]. These facts are noteworthy, as it has already been demonstrated that HOBt has a catalytic effect in the coupling mechanism [20,24]. Considering this, a critical reevaluation of reaction parameters for EDC/HOBt mediated conjugation of CS becomes necessary.

## 2. Materials and Methods

Fungal CS was supplied from KitoZyme (KiOnutrime-CsH, Herstal, Liege, Belgium) and was specified by its producer with a purity of >80% and a degree of acetalization (DA) of 0–30%. Most reagents used for modification of CS were obtained from Sigma Aldrich (St. Louis, MO, USA). These include methacrylic acid (MA, 97%), 3-(2-furyl)propionic acid (Fu, 97%), 3-maleimidopropionic acid (Mal, 97%) as well as 1-hydroxybenzotriazole (HOBt, >97%). 4-azidobenzoic acid (Az, >97%) was supplied by TCI Ltd. (Tokyo, Japan), whereas 1-ethyl-3-(3-dimethylaminopropyl) carbodiimide (EDC, 99%) was supplied by Carbolution chemicals GmbH (St. Ingbert, Germany). All reactions were conducted in inhouse produced Millipore water and dimethyl sulfoxide (DMSO, >99%), which was purchased from Fisher BioReagents (Pittsburgh, PA, USA). The NMR solvent for all experiments was obtained from Deutero GmbH (Kastellaun, Germany) as a premix of deuterated hydrochloric acid (DCl) dissolved in deuterium oxide with 0.03% of sodium trimethylsilylpropanesulfonate (DSS) as internal reference. Sodium hydroxide (NaOH, >99%), sodium chloride (NaCl, >99.5%) and hydrochloric acid (HCl, 37%) that were used for pH-adjustment and dialysis medium were obtained from Fisher Scientific U.K. Limited (Loughborough, United Kingdom), Carl Roth GmbH + Co. KG (Karlsruhe, Germany) and VWR International GmbH (Darmstadt, Germany), respectively. Cellulose dialysis membranes (molecular weight cutoff = g/mol) were obtained from Carl Roth GmbH + Co. KG and used for all purifications of CS.

The pKa of HOBt and all carboxylic acids used for conjugation with CS were determined according to a modified procedure from Pilarski et al. [27]. For this, titration of a solution containing the sample with a concentration of 0.1 mol/L in a DMSO/water mixture that was also used during conjugation (77 vol% DMSO) against 0.05 mol/L NaOH in the same mixture was used. The volume of titrant at the point of inflexion of the resulting curves, divided by two, was input into a linear fit of the initial straight part of the respective curve to calculate the pKa of a sample. All experiments were performed in triplets and the data shown represent the mean of these three experiments. 

To ensure sufficient starting material quality a purification of the CS was conducted following a modified protocol of Gan et al. [28]. For this purpose, CS (3.0 g) was stirred for 2 h at 70 °C in sodium hydroxide solution (NaOH, 50 mL, 1.0 mol/L). The resulting suspension was filtered and washed with deionized water until neutral pH was reached. This CS was subsequently dissolved in 300 mL 0.1 M acetic acid and insoluble fractions were filtered off. After dialysis against three times 20 L aqueous NaCl (0.1 M) and seven times 20 L deionized water, the purified CS was lyophilized. FT-IR and ^1^H-NMR were used for determination of DA and to identify the purified CS. Differential scanning calorimetry (DSC) was used for determination of remaining water and elementary analysis (EA) was used for quantification of chloride impurity. In summary, purified CS was obtained with a purity of 81.7% and a DA of 10%. It should be noted that remaining water and chloride impurities are a common problem when using dialysis and lyophilization for purification of CS. These impurities do not affect the reaction, but result in a systematic error in stoichiometry. The determined purity value was therefore used to correct the coupling efficacy (CE) from all conjugation reactions of CS-Az and other CS derivatives, as it was not accounted for during synthesis preparation at the start. SEC was used for determination of molecular weight distribution of purified CS. A Mn of 1.20 × 10^5^ g/mol, a Mw of 3.92 × 10^5^ g/mol, and a dispersity of 3.26 for purified CS resulted from SEC analysis. Further information of all characterizations can be found in the Supporting Information.

The main focus of this study was an optimization of the amide coupling reaction of free amine groups of CS, mediated by EDC and HOBt. All modification reactions of CS with carboxylic acid containing substituents, were conducted in sets of seven flasks per time. For this a 3D-printable attachment for standard laboratory stirrer plates, named “hepta-stirrer”, was developed. This device consists of seven inlets for standard 100 mL flasks and uses the over-extending rotating magnetic field of standard laboratory stirrers. (Build instructions and printable SLT-data can be found in Supporting Information (SI)) This approach enabled high reaction throughput and minimized differences in laboratory conditions within a set of reactions. For some sets, pH value was recorded over the full course of the reaction, using seven custom-made low-cost laboratory measurement devices (LabPi), each equipped with a temperature and pH-sensor [29]. Images of this setup and a typical reading during a reaction are shown in Supporting Information.

Basic reaction sequence and set of starting parameters were based on the work of Matsumoto et al. [6]. Synthesis steps of all experiments were conducted according to the following sequence: First, CS and HOBt were dissolved in Millipore water at ambient condition for 24 h. After this the desired carboxylic acid, for example the model substance 4-azidobenzoic acid (Az) dissolved in DMSO, was added. The starting pH was manually adjusted for some sets of reaction at this point using aqueous solutions (0.1 mol/L) of either NaOH or HCl. The actual coupling reaction was started subsequently by adding EDC dissolved in aqueous hydrochloric acid (pH = 4.2). This reaction mixture was stirred for 24 h at ambient laboratory conditions and subsequently purified by dialysis and lyophilization. For dialysis, the reaction solution was placed in cellulose membrane tubes from Roth (molecular weight cutoff 10,000–14,000 g/mol), and subjected to three times 20 L of each: NaCl (0.01 mg/mL) in 1 wt.% HCl, 1 wt.% HCl (without NaCl) and deionized water. Varied parameters include ratio of molar equivalents of HOBt to CS repetition units, amount of DMSO in reaction mixture, nature and amount of coupling reagent as well as the starting pH value. The equivalent of CS was calculated by the ratio weighted dry mass and average molecular weight. The latter was determined with DA value of purified CS, as calculated from the ^1^H-NMR-spectrum. Main experimental feedback was CE, which is defined here as ratio of theoretical maximum to final DS of a given reaction. DS is defined as molar ratio of reacted GlcN repetition units to all, GlcN and GlcNAc, repetition units from CS. The above determined purity value of CS was used here for correction of weighted CS during calculation of GlcN and GlcNAc, repetition units.

All CS derivatives produced in this way were characterized using ^1^H-NMR- and FTIR-spectroscopy (for examples spectra see Supporting Information). ^1^H-NMR was performed with an *AVIII-300* spectrometer from Bruker Corporation (Billerica, MA, USA). For this, all samples were dissolved in premixed DCl in D_2_O and DSS as internal reference. FT-IR of all CS derivatives were recorded on a *Nicolet IS50 Advance* FT-IR spectrometer from ThermoFisher Scientific (Waltham, MA, USA). A total of 64 scans with spectral resolution of 1 cm^−1^ were accumulated for each spectrum. Resulting spectra and a comprehensive table of peaks can be found in Supporting Information. 

## 3. Results and Discussion

Chitosan can be easily modified by attaching chemical groups to the amino groups of the glucosamine units in the backbone [2,3,4,5,6,7,8,9,10,11,12,13,14,15]. As already pointed out, the carbodiimide mediated amidation reaction with EDC/HOBt is a very attractive synthetic pathway for such a chitosan functionalization, which however is often associated with an insufficient degree of substitution (DS), low coupling efficacy (CE) and undesirable high reagent consumption [2,3,4,5,6,8,9,10,11,15,19]. The conjugation of chitosan (CS) with 4-azidobenzoic acid (Az) to form photoreactive arylazide chitosan (CS-Az) was used here as a model system for understanding key challenges in optimization of carbodiimide mediated polymer analogous reactions. The resulting modified CS is also of interest for generation of various water-resistant nanostructures, as it can be rapidly crosslinked on demand. Indeed, irradiation of CS-Az with UV-light results in highly reactive but also chain-bound nitrene species that react quickly with C-H- and N-H-groups of adjacent molecules, thus forming a network [17,18]. This has already been used e.g., for generation of thin coatings on polyimide surfaces for neuronal implants, increasing hydrophilicity and biocompatibility, while preventing undesirable cell growth [16]. For this, Hadler et al. have produced CS-Az by using DMT-MM as coupling agent. The coupling efficacy (CE) of this method was approximately 50%, meaning that only half of all desired Az molecules were incorporated as CS side chains. This complicates the generation of CS-Az with accurate DS. For some applications, e.g., synthesis of graft polymers suitable for polymersome formation, it is important to tailor DS. Indeed, Bourgat et al. were able to prepare polymersomes comprised of a CS-graft-polycaprolactone copolymer with very specific number of hydrophobic components in the polymer chains [30]. To enhance accuracy, as well as for economic and ecologic aspects, it is crucial to exert tight control over the synthesis and to reach a maximum CE close to 100%. 

Matsumoto et al. already proposed a method for conjugation of hydrophobic components, such as Maleimides, onto shrimp CS [6]. This method uses EDC as coupling reagent and HOBt as an additive to enhance the reaction rate and to minimize the formation of side-products. Both reagents, EDC and HOBt, were used in stoichiometric amounts relative to chitosan. DMSO was used as a cosolvent together with water in a 50 vol% mixture to increase the solubility of the reaction intermediates and subsequently the yield of the reaction. Furthermore, it was hypothesized that a large amount of HOBt formed a complex with CS and enhanced its solubility [6]. The conditions used resulted in high CE (close to 100%) when a DS up to 20% was targeted. 

**Figure 1:**
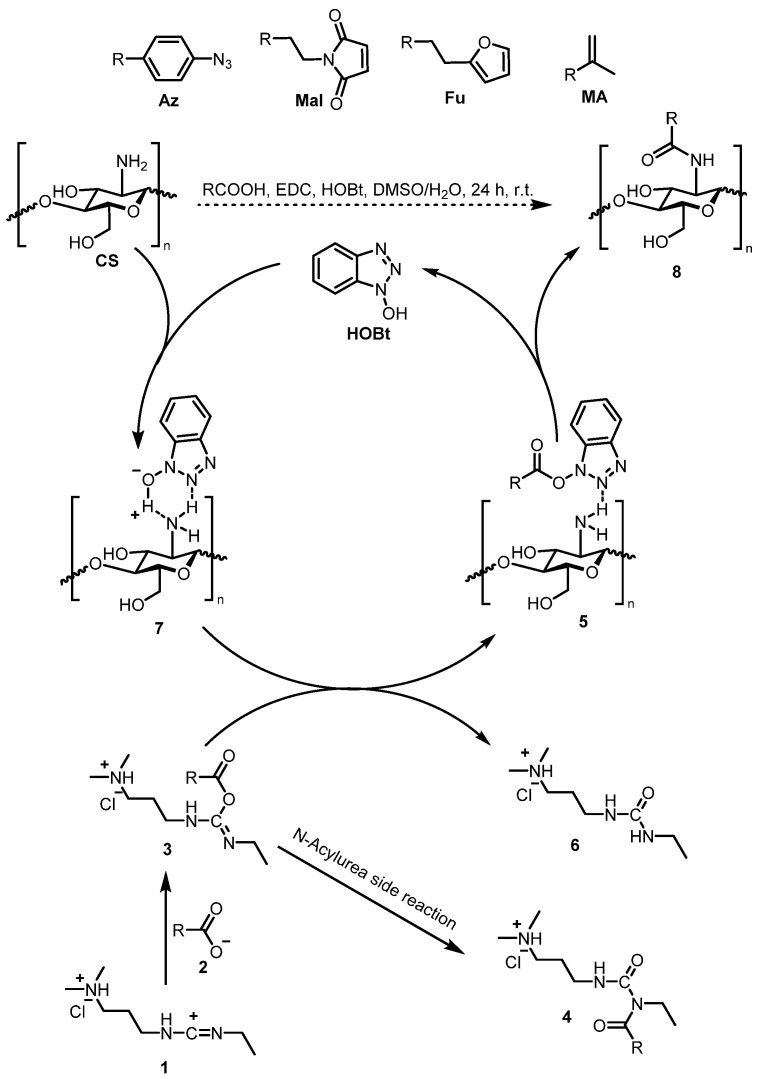
Cyclic form of reaction mechanism from EDC/HOBt mediated amide coupling for conjugation of different carboxylic acids (Az, Mal, Fu and MA) to CS, rearranged, redrawn and complementedfrom literature [11,24].

In preliminary experiments, similar conditions have been used to synthesize CS-Az with targeted DS between 6% and 35%. In these experiments unexpected low CE for all desired DS have been found. Using the same ratio of reactants and the exact experimental procedure as Matsumoto [6], 19.8% was the highest CE that could be reached (data not shown). The only differences between these experiments and those done by Matsumoto et al. [6] was the use of a different carboxylic acid (Az instead of Mal) and a different biological source of CS. To address sustainability aspects, CS extracted from fungal biomass was favored in this study. Nevertheless, the studied fungal CS with a DA of 10% and an average molecular weight of Mn of 1.20 × 10^5^ g/mol with a polydispersity of 3.26 is similar to that used by Matsumoto et al. [6]. Obviously, the use of a different carboxylic acid with different properties, such as dissociation constant, pKa, and solubility, affects the reaction. However, such a drastic decrease of CE was unexpected and suggested that other parameters needed to be addressed as well. Furthermore, Matsumoto et al. used stoichiometric amounts of HOBt, [6] although it has been reported to have a catalytic role in the mechanism [23,24]. Overall, the limitations of literature conditions [6] for conjugation of CS with Az demonstrate the need for critical reevaluation and optimization of reaction parameters. The mechanism of the conjugation reaction as described in the literature [11,24] is shown as a catalytic circle in Figure 1 and briefly discussed below with special emphasis on the role of HOBt and other parameters.

As described by Cox et al. the overall reaction of any EDC/HOBt mediated amide coupling is governed by the rate-determining reaction between the doubly protonated form of EDC **1** (see Figure 1) and a deprotonated carboxylic acid **2** [24]. Thus, EDC must be protonated both at the carbodiimide carbon and the terminal tertiary amine to react with the deprotonated carboxylic acid **2** and to form the desired O-acylisourea **3**. For the sake of brevity, both cyclic tautomeric forms of EDC are not shown here, but are included in this reaction step as well [31]. Cox et al. observed a maximum conversion rate at pH = (pKa_(carboxylic acid)_ + pKa_(EDC)_)/2. As an example, acetic acid and EDC with a pKa of 4.7 and 3.1, respectively, have an optimal pH of 3.9 for conjugation in water [24]. A rapid decrease of conversion at pH > 4.7 due to increased deprotonation of **1** and at pH < 3.1 due to the protonation of **2** has been used for explanation of the observation [24]. In this respect, every factor that influences the pKa of the reagents such as EDC, HOBt, or the carboxylic acid affects the outcome of the reaction significantly. The use of DMSO as cosolvent, represents an example for such an important factor. Another key element in the reaction mechanism is the use of a benzotriazole additive such as HOBt, which suppresses the formation of N-acylurea **4** by rapidly reacting with **3** to form the activated ester **5** and the urea form **6** of EDC. In fact, the formation of **4** is undesirable because it leads to unproductive consumption of **2**. During the conjugation of CS as a special case, HOBt and CS are thought to form a water-soluble complex **7**, generated by hydrogen bonds between HOBt and amino groups of CS. [11] This implies that the active ester **5** is directly in the vicinity of the primary amine from GlcN, effectively bringing all three reaction partners close to each other. Finally, the rearrangement of **5** leads to the desired CS derivate **8** and the release of HOBt. In summary, the success of the above reaction is strongly dependent on the three experimental parameters HOBt and DMSO concentration as well as pH, which were therefore studied in a first attempt to optimize the coupling reaction.

**Figure 2:**
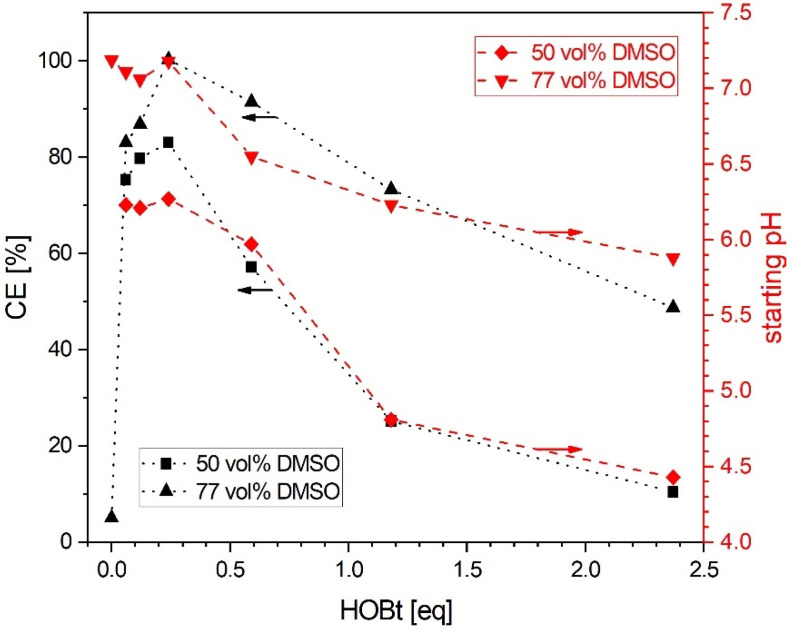
Influence of HOBt and DMSO on CE and pH of conjugation of CS and Az. The pH value was measured directly after adding 0.95 equivalents of EDC as the final reagent to each batch. The lines connecting data points are a guide to the eye.

To optimize the CE of the reaction, the quantity of HOBt was systematically varied from catalytic (0.06–0.24 equivalents) to stoichiometric (0.59–2.37 equivalents) amounts (based on 1 equivalent CS) at two concentrations (50 vol% and 77 vol%) of DMSO. The starting pH, representing the pH value after adding 0.95 equivalents of EDC as final reagent to a batch, was also measured as a third parameter. One of the most noteworthy results, observable in Figure 2 is that CE was significantly increased when catalytic instead of stoichiometric amounts of HOBt were used. Given that most published conjugation experiments use stoichiometric amounts of HOBt [2,3,4,5,6,8,9,10,11,15,19], our finding represents an unexpected result at first sight. Indeed, a maximum of 81.3% CE at 0.24 equivalent of HOBt was found at 50% DMSO, already representing a drastic increase compared to initially tested conditions taken from literature [6]. Moreover, the CE can also be correlated with the starting pH. Accordingly, using stoichiometric amounts of HOBt, resulted in a decrease of starting pH similar to CE. Rather than simply acting as a catalyst and increasing reaction rates, it seems that HOBt also decreases pH of the reaction mixture and thereby CE as well. 

Repeating the experiments at higher DMSO content of 77 vol% resulted in a similar trend. At higher DMSO content, CE and starting pH were always higher compared to lower DMSO contents. Indeed, a maximum of 100.0% CE at 0.24 equivalent of HOBt in 77 vol% DMSO was found, indicating full conversion of Az to CS-Az. It should be noted that accuracy of CE determination of the reaction is limited due to variances in determination of DS of CS-Az via ^1^H-NMR. Other reasons are cumulated errors in impurity analysis via EA and DSC of purified CS, which were used to calculate the CE after synthesis (see Supporting Information). Nevertheless, CE and pH values also decreased at higher amounts of HOBt, similar to the situation in 50 vol% DMSO. A minimal CE of 48.6% at 2.37 equivalents of HOBt was found in one case. We hypothesize that a decreased acidity of HOBt (and other reagents) at higher DMSO content is responsible for the higher pH and CE values. In fact, HOBt is weakly acidic, with a pKa determined by titration in 50 and 77 vol% DMSO of 5.08 +/− 0.07 and 6.67 +/− 0.03, respectively. Additionally, CE was found to be drastically diminished to 5.2% without HOBt, thus indicating the general importance of HOBt for the reaction. We therefore hypothesize that the starting pH can be used directly for optimization of CE, independent of HOBt and DMSO content. This would explain above results and show an easy opportunity for optimization.

**Figure 3:**
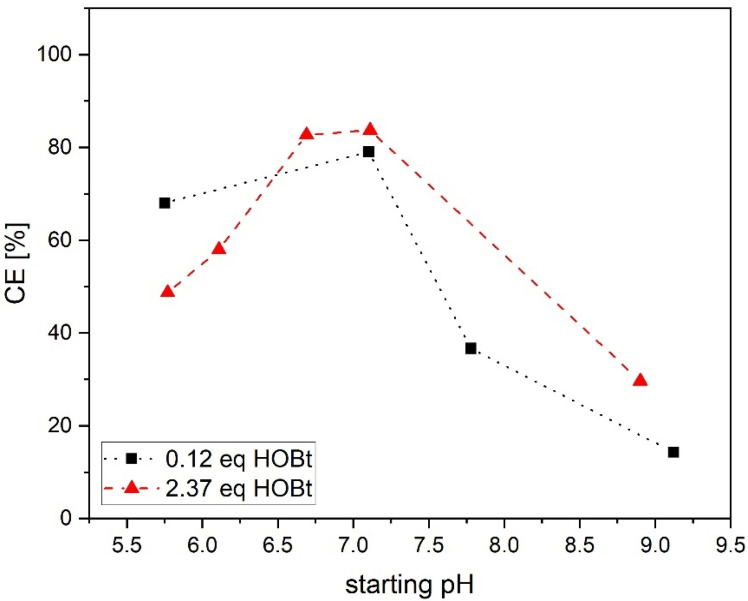
Differentiation between influence of HOBt and starting pH on CE of conjugation of CS and Az in a mixture of 77 vol% DMSO and water with 0.95 equivalents of EDC. The lines connecting data points are a guide to the eye.

To validate the hypothesis and to differentiate between the influence of pH and HOBt on CE of the reaction another set of experiments was carried out. For both DMSO contents high CE at 0.12 equivalent HOBt and low CE at 2.37 equivalent HOBt were found in above experiments. These two extreme values were therefore used in two sets of reactions at a fixed DMSO content of 77 vol% and 0.95 equivalents of EDC. Here the starting pH was adjusted manually with 0.1 M NaOH or 0.1 M HCl directly before addition of EDC and varied between 5.8 and 9.0 while the amount of HOBt was kept fixed at 0.12 and 2.37 equivalents, respectively. In both cases, a similar maximum CE was found at a pH of 7.1 (Figure 3) This indicates the strong influence of pH on CE and shows that it is not necessary to use stoichiometric or higher amounts of HOBt to enhance the reaction.

As mentioned above, Cox et al. have observed a maximum conversion rate at pH = (pKa_(carboxylic acid)_ + pKa_(EDC)_)/2 for the reaction of acetic acid and EDC in water (acetic acid and EDC with a pKa of 4.7 and 3.1, respectively) [24]. This optimal pH represents a situation where equal concentrations of protonated EDC and deprotonated carboxylic acid in the reaction mixture are expected. This should be clearly beneficial for maximum conversion rate, resulting in high CE [24]. However, in the case of the present study, Az was conjugated to CS in a mixture of 77 vol% DMSO and water. As there was no data available in the literature, pKa of Az was determined by titration to be 7.71 +/− 0.09 in this mixture. In fact, solvents such as DMSO have been shown to shift pKa of some groups of molecules, including carboxylic acids, to rather high values, while leaving others, such as tertiary amines (e.g., EDC, with a pKa of 3.1) relatively unchanged [24,25]. It has been shown for example that pKa values from 12 tested carboxylic acids measured in pure DMSO increased by a median of 7.2 when compared to the same compounds in water [25]. It should also be noted that the presence of water in mixtures of such solvents reduces this effect somewhat [24]. Therefore, our empirically determined pKa value in 77 vol% DMSO and water is very plausible. In conclusion, the determined pKa value of Az can be used in above formula to calculate a theoretical optimum pH at 5.4. This, however, is in contradiction with our findings of low CE at pH around 5.8 and an empirically observed optimal pH region between pH 6.7 and 7.4, which is well above the theoretical optimum. It seems that optimal pH is asymmetrically shifted close to Az and not exactly in between pKa of EDC and Az. At the observed optimal pH region around 7.0 this would result in a very low population of protonated EDC **1** and a drastically higher population of deprotonated Az **2**. Both conditions are not optimal for a second-order kinetic from a mechanistic perspective. One possible explanation lies in the fact that the above formula for optimal pH is used for aqueous buffer systems, while we conducted the reaction at high volume fractions of DMSO. Solvents similar to DMSO in respect to carboxylate solvation and dielectric constant, such as *N*-Methyl-2-pyrrolidone (NMP) or Dimethylformamide, have already been shown to not only to shift pKa but also to influence the intrinsic reactivity of reagents, especially carboxylic acids [24,25]. For example, Cox et al. determined a rate constant for an EDC mediated amide formation in NMP that was about 10^5^ times higher than comparable reaction rates of EDC and acetic acid in water [24]. Despite the drastically increased intrinsic reaction rate, the actual observed rate constant was again comparable with that in water. It was argued that shifted pKa value of carboxylic acid and resulting lower population of reactive species are the reason for that [24]. We probably observed a similar situation as DMSO is comparable with NMP in respect to solvent parameters such as dielectric constant and the reduced solvation of the carboxylate anion compared to water [24]. Actually, the experiments summarized in Figure 2 show already such an effect. Here the use of a solvent system with less DMSO (e.g., 50 vol%) resulted in lower CE and pH and probably a lower intrinsic reactivity of the reagents. 

For testing applicability of the parameters experimentally found, a broader set of carboxylic acids, namely methacrylic acid (MA), 3-(2-furyl)propionic acid (Fu), 3-maleimidopropionic acid (Mal) as well as 4-azidobenzoic acid (Az) (see Figure 1) were conjugated to CS following the same procedure as described above. The optimized parameters, 0.12 equivalent HOBt, 77 vol% DMSO content and a manually adjusted starting pH of 7.0 +/− 0.1 were used to generate CS derivates with a theoretical maximum DS of 35.5%. Additionally, all pKa values of the three new carboxylic acids in the reaction solvent system (77 vol% DMSO) were determined. The results are summarized in Table 1.

Production of CS-Az, CS-Fu, CS-MA and CS-Mal could be achieved with high CE with the corresponding carboxylic acids having a pKa in the region between 7.71 and 8.54 in 77 vol% DMSO. CS-Mal for example was obtained with a high DS of 30.1% at low HOBt content. Compared to the results of Matsumoto et al. [6], this demonstrates again that stoichiometric amounts of HOBt are not necessary for efficient coupling of carboxylic acids to CS. In conclusion, all four functional derivatives were obtained with high DS and with CE ranging from 80.7 to 90.2%. This highlights the wide applicability of our method and the possibility to optimize CE by adjusting the pH of the reaction mixture. We expect similar results for other functional molecules, which will render the optimized method very useful for all fields of chitosan research.

## 4. Conclusions

Our findings demonstrate the importance of using optimized parameters for functionalization of chitosan via carbodiimide chemistry. The usually proposed stoichiometric use of HOBt for example caused low coupling efficacy (CE), while using only catalytic amounts of HOBt for modification of CS with Az resulted in significantly higher CE. This is in accordance with the mechanism and underlines the catalytic nature of the hydroxy benzotriazoles in the coupling. However, we found that the pH has a more decisive role for CE. In this respect also the role of DMSO in the reaction must be reconsidered, because the pKa of the reagents is changed in DMSO water mixtures compared to pure water. By identifying an optimal pH-value and carefully controlling the starting pH, we were able to demonstrate, for the first time, maximum coupling efficacy for synthesis of CS-Az with a degree of substitution of 35.5%. Furthermore, the optimized conditions enabled significant reduction of reagents consumed, rendering the method ecologically and economically superior for larger scale production of various CS derivatives with defined DS. This will in turn enable their widespread application as biologically active coatings, functional nanomaterials such as nanofibers or drug-delivery systems.

## Figures and Tables

**Table 1:** Experimentally determined pKa values of different functional carboxylic acids in 77 vol% DMSO and water as well as DS and CE results for conjugation of these reagents to CS under the same set of optimized parameters.

Carboxylic Acid Used for Conjugation Reaction	Experimental pKa of Carboxylic Acid	Resulting CS Derivate	DS [%]	CE [%]
4-azido benzoic acid	7.71 +/− 0.09	CS-Az	28.7	80.7
3-(2-furyl) propionic acid	8.50 +/− 0.08	CS-Fu	29.2	82.2
methacrylic acid	8.54 +/− 0.05	CS-MA	32.0	90.2
3-maleimido propionic acid	8.78 +/− 0.04	CS-Mal	30.1	84.8

## Data Availability

The data that support the findings of this study are available from the corresponding author, upon reasonable request.

## Supplementary Materials

The following are available online at https://www.mdpi.com/article/10.3390/polym13162702/s1, Figure S1: Image of all seven 3D-printable pieces (printable files 1, 2 and 3) as well as build instructions for hepta-Stirrer. Figure S2: Set of seven conjugation reactions in round flasks, sitting in 3D-printed hepta-stirrer. Figure S3: Results from SEC measurement of purified CS. Figure S4: Results from DSC water content analysis of purified CS. Table S1: Experimental and theoretical elemental composition of purified CS with DA of 10.3% and water content of 12.2%, determined by ^1^H-NMR and DSC experiments, respectively. Figure S5: ATR-IR spectra of: (a) purified CS as well as all produced CS derivatives: (b) CS-Fu, (c) CS-Az, (d) CS-MA and (e) CS-Mal. Figure S6: ^1^H-NMR spectra of: (1) purified CS as well as all produced CS derivatives: (2) CS-MA, (3) CS-Mal, (4) CS-Fu, (5) CS-Az. Table S2: Comprehensive list of all relevant ^1^H-NMR and FT-IR signals from synthesized CS derivatives concerning conjugated functional groups.
