# Supplementary material for: Optimization of Critical Parameters for Carbodiimide Mediated Production of Highly Modified Chitosan
Source: Polymers (Basel). 2021 Aug 13;13(16):2702. doi: 10.3390/polym13162702 (PMC8399066; doi:10.3390/polym13162702)
Supplement: Supplementary file 1 [file polymers-13-02702-s001.zip › 20210719 Supporting Info Optimization of critical parameters for carbodiimide mediated production of highly modified chi.pdf]

Supporting information:

<u>Building instructions for the hepta-Stirrer:</u>

For the "hepta-Stirrer", three printable SLT data files were designed (hepta-Stirrer 1, 2 and 3, see Figure S1). These files can be found in the electronic section of SI. The first two pieces (hepta-Stirrer 1 and 2) need to be printed out thrice, while the middle piece (hepta-Stirrer3) is only required once. We achieved reasonable print quality using a MK3 3D-printer from PRUSA RESEARCH (Prag, Czech Republic) with white PLA filament at a layer thickness of 0.2 mm. For assembly, three pieces of 1 were set on a plane and connected together via three pieces of 2. The middle piece 3 needs to be inserted last and stabilizes the build (Figure S1). The hepta-Stirrer is designed to fit a laboratory stirrer plate with 135 mm diameter from IKA (Staufen, Germany).

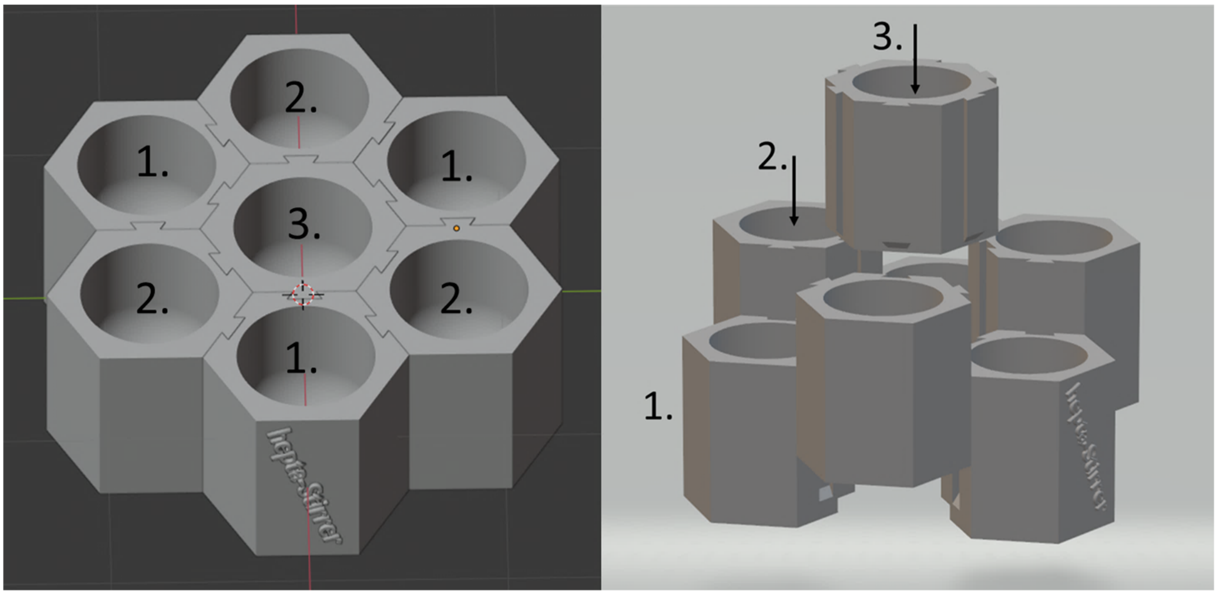

Figure S1: Image of all seven 3D-printable pieces (printable files 1, 2 and 3) as well as build instructions for hepta-Stirrer.

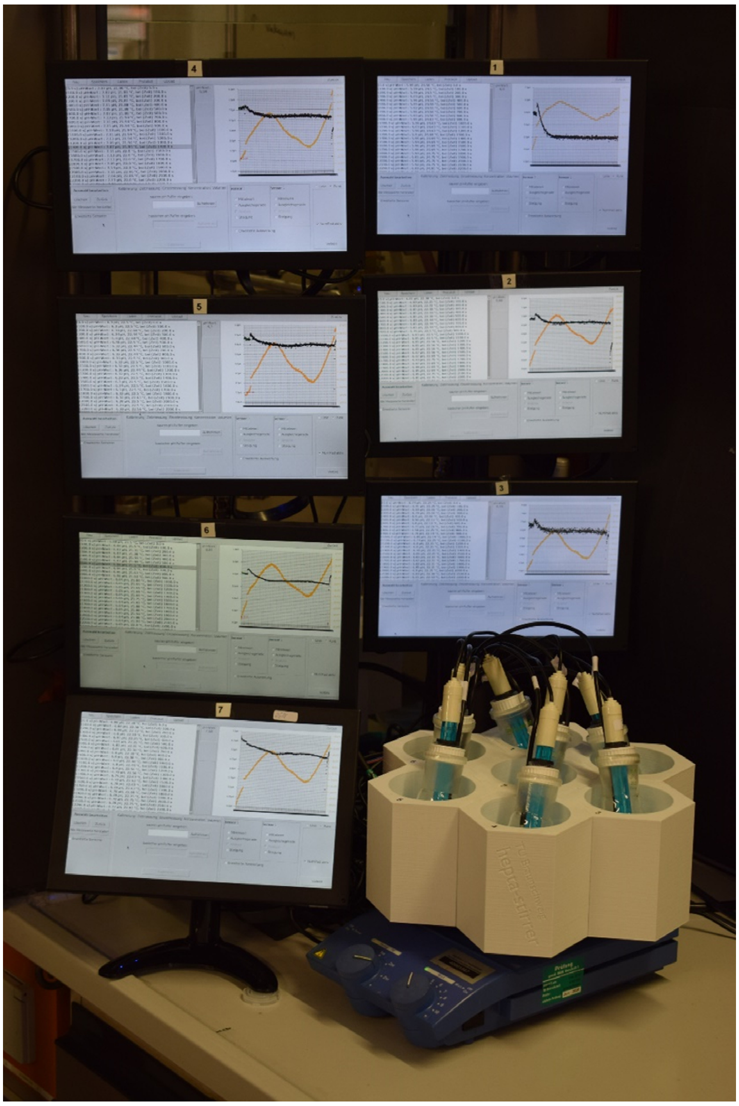

Figure S2: Set of seven conjugation reactions in round flasks, sitting in 3D-printed hepta-stirrer and equipped with pH electrodes and temperature sensors as well as LabPi's showing data from complete reaction time.

<u>Characterization of purified CS and analysis of remaining impurities:</u>

Purified CS was characterized via ^{1}H-NMR and FT-IR spectra. Signals at 2.05 (3H, acetyl from GlcNAc), and 3.01 – 4.14 (6H, ring backbone from GlcN and GlcNAc) can be easily identified. Beside these two important signals a huge water signal, overlaying the anomeric proton signals of GlcN and GlcNAc, can be found. This indicates water impurities in CS. In FT-IR spectra typical peaks of CS can be found: 3354 cm^{-1} (ν(O-H) and ν(N-H)), 2921 cm^{-1} (ν(C-H)), 1633 cm^{-1} (amid I and amine), 1529 cm^{-1} (amid II) 1254 cm^{-1} (fingerprint), 1066 cm^{-1} (ν(C-O)). (For respective spectra see Figure S5 and Figure S6.)

SEC measurements were performed on a *SECcurity*^{2} instrument equipped with a *SECcurity*^{2} vacuum degasser and a *SECcurity*^{2} *TCC6500* column oven from PSS GmbH (Mainz, Germany). An *Infinity 1260 I* isocratic pump, an *Infinity 1260 I* refractive index detector (RI) and an *Infinity 1260 II* diode array detector (DAD) from Agilent Technologies, Inc. (Santa Clara, CA, USA) were also used. For SEC analysis of chitosan, a sample of each, purified CS and CS-Az with low DS (10 %) was dissolved at a concentration

of 1.0 g/L in an aqueous buffer system comprised of 0.1 M NaCl and 0.1% TFA at pH = 1.9. Ethylene glycol was used as an internal standard in the buffer system at a concentration of 2.000 g/L. Samples with DS higher than 10 % were not completely soluble in the used buffer system and thereby excluded from analysis. A volume of 100 µL of the samples was passed over a Novema max column from PSS GMBH at a flow rate of 1 mL/min and a temperature of 40 °C. The signal from the RI in combination with ten pullulan standards from 7.080 $*10^5$ g/mol to 3.42 $* 10^2$ g/mol was used to calculate the molecular weight distribution and is shown in Figure S3. The results for CS-Az are very similar to that of purified CS, indicating no significant changes of CS chains during synthesis. Also, another very similar set of results was found for CS-Az when using the DAD signal of the arylazide at 285 nm, indicating homogenous incorporation of Az over complete molecular weight distribution (data not shown).

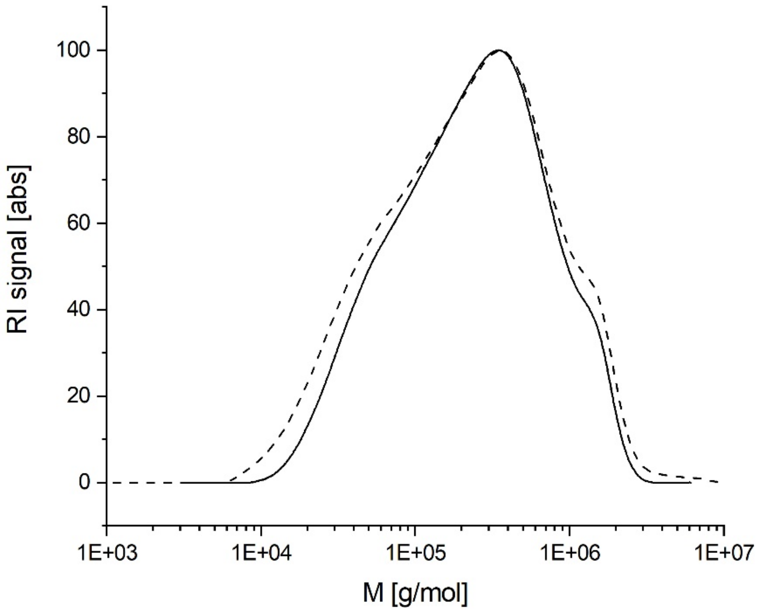

Figure S3: Results from SEC measurement of purified CS (solid line) with Mn = 1.20 $*10^5$ g/mol, Mw = 3.92 $*10^5$ g/mol, Đ = 3.26 and CS-Az with a DS of 10 % (dashed line) with Mn = 9.74 $*10^4$ g/mol, Mw = 4.23 $*10^5$ g/mol, Đ = 3.87.

Differential scanning calorimetry (DSC) measurements for determination of water content in purified CS were performed with a *DSC 204* from NETZSCH GMBH & CO. KG (Selb, Germany). Briefly, 10 mg of sample were sealed and subjected to two measurement cycles, beginning with a heating step (-20 °C to 150 °C, speed of 10 °C/min), an adjacent isothermal step (150 °C for 20 min) and ending with a cooling step (150 °C to -20 °C, speed of 10 °C/min). Remaining water content was calculated by difference of sample weight before and after this treatment. The first heating curve of CS sample shows an endotherm signal for water evaporation with a peak at 106.8 °C of 0.9756 W/g and an area of 239.3 J/g, integrated with horizontal baseline, starting from left edge (see Figure S4). The second heating curve shows no such peak, indicating complete evaporation during first heating. When initial sample weight of 8.195 mg and enthalpy of evaporation of 2257 J/g from water are taken into account, it can be calculated that 0.9 mg of water has been evaporating during the first heating cycle. This estimate correlates well with

gravimetric water loss of 0.999 mg during the complete procedure, which results in a gravimetrically determined water content of 12.2 % in purified CS.

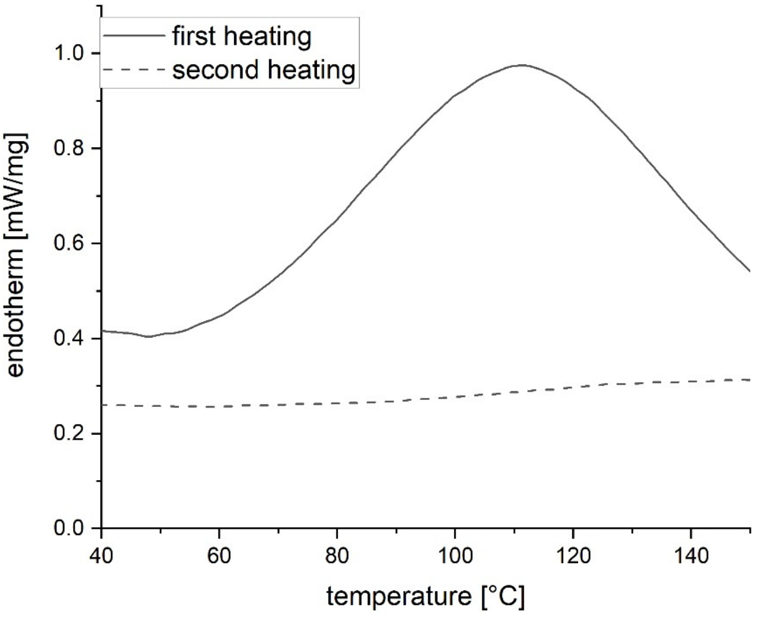

Figure S4: Results from DSC water content analysis of purified CS.

Preliminary experiments suggested Cl as a second impurity beside water in our CS starting material. For quantification, EA experiments were performed on a *Flash EA 1112* from THERMOQUEST ITALIA S.P.A. (Milano, Italy). Indeed, a chlorine content of 6.11 +/- 0.08 % was found in the resulting data. Theoretically expected values for C, H, N, and S were calculated, factoring in the water and chloride content as well as DA to compare with our data (see Table S1).

Table S1: Experimental and theoretical elemental composition of purified CS with DA of 10.3 % and water content of 12.2 %, determined by ^{1}H-NMR and DSC experiments, respectively. Mean and standard deviation were calculated from two EA measurements. * = determined from experimental value.

element	expected elemental content of CS [%]	experimental values of purified CS [%]
C	36.75	36.95 +/- 0.04
H	6.95	6.81 +/- 0.01
N	6.95	6.52 +/- 0.05
S	0.00	0.00 +/- 0.00
Cl	6.11*	6.11 +/- 0.08

It can be concluded that CS was obtained with a purity of 81.7 %. Both impurities probably arise from dialysis against aqueous 0.1 M NaCl and incomplete lyophilization. Therefore, we hypothesize that Cl is present as chloride ions. Generating CS without these impurities would need a different purification method. For the case of bound water, it can be noted that chitosan is highly hygroscopic and also able to quickly reabsorb water from the atmosphere, making accurate weighing and complete drying hard to accomplish. These uncertainties in combination with limited accuracy of NMR results can result in a

systematic error in determination of CE of the conjugation reactions, as the purity was used for calculation of CE.

Characterization of all synthesized CS derivatives:

Besides from aforementioned ^{1}H-NMR and FT-IR signals from purified CS all produced derivates clearly show signals that are specific for the used carboxylic acid containing functional group. These relevant signals are listed in Table S2 and indicate successful conjugation in all cases. For further information an example FT-IR and ^{1}H-NMR spectra of CS, CS-Az, CS-Mal, CS-MA and CS-Fu is given in Figure S5 and Figure S6, respectively.

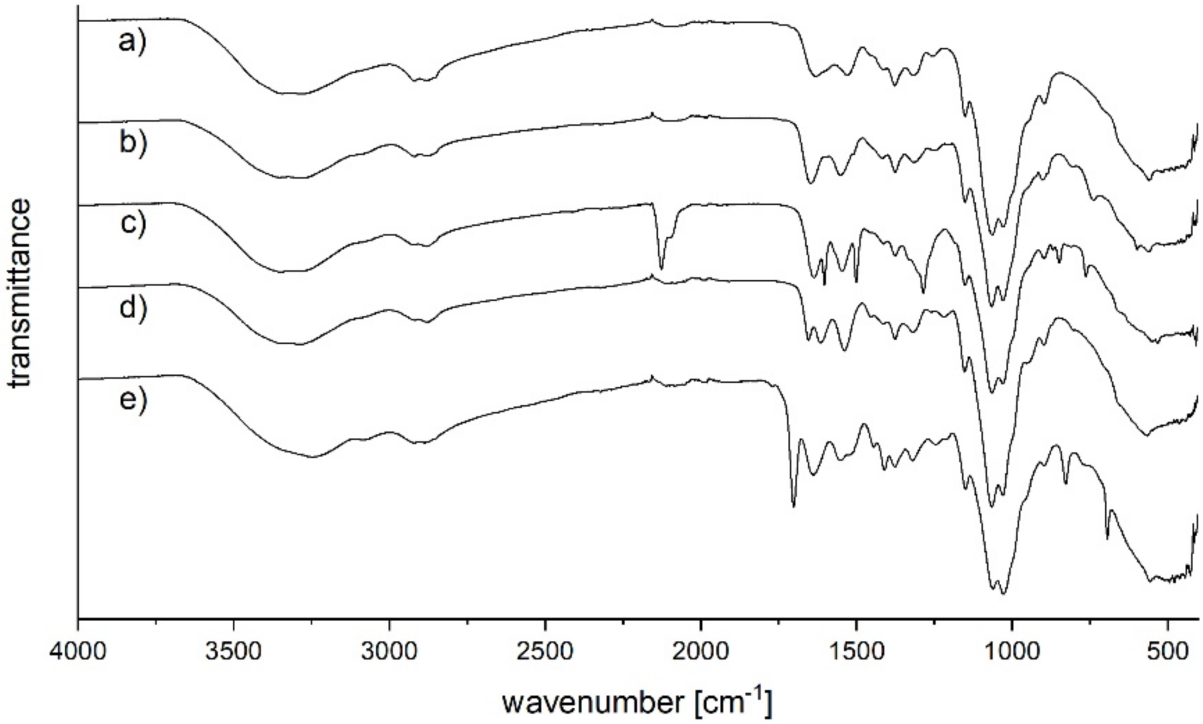

Figure S5: ATR-IR spectra of: a) purified CS as well as all produced CS derivatives: b) CS-Fu, c) CS-Az, d) CS-MA and e) CS-Mal.

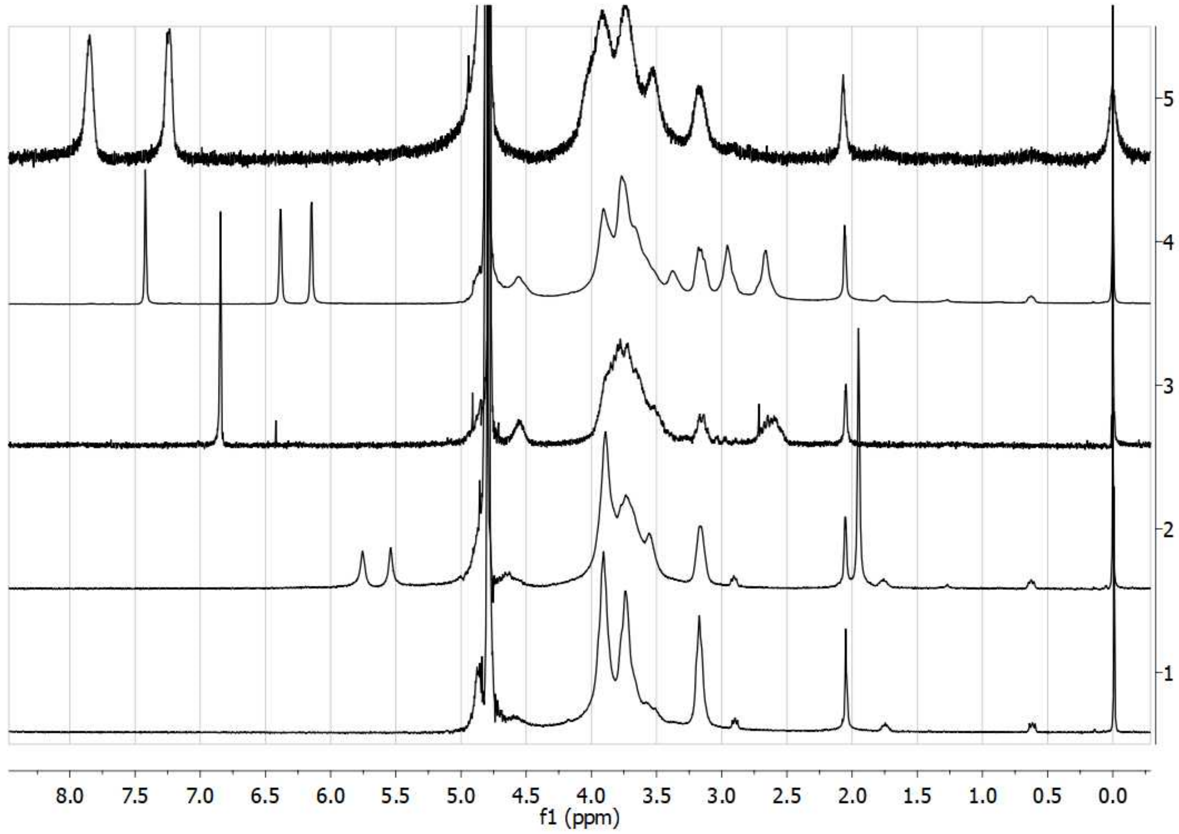

Figure S6: ^{1}H-NMR spectra of: 1) purified CS as well as all produced CS derivatives: 2) CS-MA, 3) CS-Mal, 4) CS-Fu, 5) CS-Az. It should be noted here that signals at 0.00, 0.63, 1.75 and 2.91 ppm can be attributed to reference compound DSS. The signal at 4.75 corresponds to water impurities from dialysis and overlays all anomeric proton signals from repetitive units of CS or derivates thereof.

Table S2: Comprehensive list of all relevant ^{1}H-NMR and FT-IR signals from synthesized CS derivatives concerning conjugated functional groups.

**CS derivate**	**^{1}H-NMR signal shifts relevant for conjugation [ppm]**	**ATR-IR signals relevant for conjugation [cm^{-1}]**
CS-Az	7.21 (2H, phenyl), 7.82 (2H, phenyl).	2127 (azide), 1641 (amid I), 1604 (arom. I), 1548 (amid II), 1501 (arom II), 1286 (amid III), 849 (fingerprint), 764 (fingerprint)
CS-Fu	2.66 (2H, aliphatic), 2.96 (2H aliphatic), 6.15 (1H, aliphatic), 6.38 (1H, aliphatic), 7.42 (1H, aliphatic).	1648 (amid I), 1553 (amid II), 811 (fingerprint), 742 (fingerprint)
CS-MA	1.95 (3H, aliphatic), 5.54 (1H, aliphatic), 5.76 (1H, aliphatic).	1655 (amid I), 1616 (double bond), 1539 (amid II)
CS-Mal	2.52 – 2.72 (2H, aliphatic), 5.57 (2H, aliphatic), 6.85 (2H, double bond).	1702 (double bond), 1640 (amid I), 1556 (amid II), 829 (fingerprint), 695 (fingerprint)
